# Design, Simulation, and Development of a BioSensor for Viruses Detection Using FPGA

**DOI:** 10.1109/JTEHM.2021.3055984

**Published:** 2021-02-01

**Authors:** M. Abdallah

**Affiliations:** SUNY Polytechnic Institute 14627 UticaNY13504USA

**Keywords:** Biosensor, impedance, nanoscale, virus testing, FPGA

## Abstract

*Objective:* Impedance based biosensing provides a unique, highly sensitive electrical approach to biomolecule detection, cell growth, and other biological events. To date, an impedance change due to the cell growth has been considered as a solution to detect some changes in a cell’s behavior. The impedance change detection is normally measured via an impedance analyzer which is expensive and also cumbersome. Rapid and definitive diagnosis of viral infections is imperative in patient treatment process. Early detection followed by appropriate lifestyle and treatment may result to a longer, healthier life. Certain patients require continues monitoring that may require regular visits to hospitals which is not practical. Therefore, a continuous home healthcare device is needed to monitor and detect any change in a patient’s health condition. *Methods & Results:* In this research, a novel sensor and healthcare monitoring system is modeled, simulated, developed, and tested to detect viruses by detecting the change in the impedance due to antibodies and antigens binding. First, COMSOL simulation tool is used to develop a model to prove the concept. The model predicts increasing impedance during functionalization of electrodes with antibodies and after antigen binding steps. Second, to understand how nanoscale electrode size and spacing would affect biosensing assay (antibody-based affinity binding of a protein antigen), a model using COMSOL is developed. Third, Field Programmable Gate Arrays (FPGA) based signal processing system is developed as well to be connected to analog to digital converter (ADC) to acquire the current and voltage readings of the sensors over time. This healthcare monitoring system is used to continuously monitoring a patient’s condition and reports any changes in the impedance readings which represents virus detection or at least change in the cell’s behavior. *Conclusions:* The proposed sensor model is simulated, tested and verified via COMSOL and the FPGA prototype is tested and it verified the COMSOL model. This work reports that the proposed sensor can be used to detect viruses via detecting a change in the impedance.

## I. Introduction

Field Programmable Gate Arrays (FPGAs) are special type of embedded systems that enable programmability with minimum design time. One of the main advantages of using FPGA is the reconfigurability in the field after fabrication; hence comes the name “field programmable” [1].

Biosensors can be used in a wide range of applications to detect cells [2], toxins [3], and to monitor diseases in early stages [4], [5]. A variety of mechanical, optical, and electrochemical concepts can be used to design and develop biosensors [6]–[8]. Electrochemical detection methods are ideal for biosensing applications, especially in the case of quick and affordable detection [8]. Electrochemical biosensors measure changes in electrical properties that result from the presence of biological moieties (whole cells, proteins, nucleic acids, and other biomarkers). Changes in the electric field at the surface of the electrodes are measured when biological moieties interact with the sensor, or within the spaces between the sensor’s electrodes [9], [10].

Sensor’s electrodes are used to detect and analyze cells or biomarkers by using multi-frequency impedance-based characterization. Detection and characterization are based on the dielectric properties of the cells/biomarkers (capacitance and conductivity). In the case of whole cells, this can lead to the detection of physiological differences between cells or changes in cell morphology or size over time [11]. They can achieve a wide range of advantages over the traditional methods such as real-time detection, label-free analysis, non-invasive sensing, ease of integration, and high throughput screening [12], [13].

Based on the space between the electrodes and the bio- or chemical-selective layer deposited on the electrodes, a sensor can detect any analyte that has a difference in dielectric constant from the background solution/medium. For resistive chemical sensors, a conductive thin layer should cover the electrodes. The thin sensing area resistance will change due to the concentration of the analyte. Randle’s model can be used to understand the electrical interactions between the electrodes, sensing layer, target cells, and surrounding solution [14].

The impedance change, due to the cell growth, has been considered as a solution to detect some changes in the cell’s behavior. It can be modeled as an equivalent circuit consisting of resistors and capacitors of both the cell culture media and the cells. To date, the impedance change detection were measured via an impedance analyzer which is expensive and also cumbersome. Other research work has been performed on the dimensions of the sensor to optimize the sensing area [15], [18].

Previously an impedance-based model was reported for cell counting using COMSOL [19]. The effect of gold nano particles on sensors were modeled using the same platform [20]. Cell growth modeling [21] and bacterial (*E. coli*) detection [22] also been reported. Even though antibody-antigen based biosensing is one of the most common ways impedance sensors have been used, there has no known COMSOL model reported on such a system.

Early diagnosis of viral infections is imperative in patient treatment process. Certain patients require continues monitoring or they are required to make regular visits to their physicians for tests. This process is very complicated and costly. Nationally, it is desired to minimize the unnecessary trips to clinics and hospitals. Therefore, a home healthcare device is needed to continuously monitor and detect any change in a patient’s health status and only if there is a need to the hospital visit, a patient must make this trip.

In this research, a novel sensor and healthcare monitoring system is modeled, simulated, developed, and tested to detect viruses by detecting the change in the impedance due to antibodies and antigens binding. The paper is organized as three main parts. First, COMSOL is used to develop a model to prove the concept of the ability to use the impedance change as an indication of a change in a human cell’s behavior. In other words, the model is simulated to prove that there is a correlation between the change in the impedance and the change in a human cell. The model predicts increasing impedance during functionalization of electrodes with antibodies and after antigen binding steps. Second, to understand how nanoscale electrode size and spacing would affect biosensing assay (antibody-based affinity binding of a protein antigen), a model using COMSOL is developed to help to optimize the sensor parameters such as the spacing between electrodes and dimensions. Third, Field Programmable Gate Arrays (FPGA) based signal processing system is developed as well to be connected to analog to digital converter (ADC) to acquire the current and voltage readings of the sensors over time. This healthcare monitoring system is used to continuously monitoring a patient’s condition and reports and change in the impedance readings which represents virus detection or at least change in the cell’s behavior.

## II. IDE Sensor Design and Simulation

The finite element analysis multiphysics simulation software COMSOL was used to simulate an impedance spectroscopy sensor. The AC-DC module of COMSOL was used to simulate the electrical response of impedance sensor, add bio-selective materials (antigens) to the sensor, and to calculate the change in the impedance upon addition of biological target elements (antibodies/IgG) to the sensor electrodes.

**FIGURE 1.**
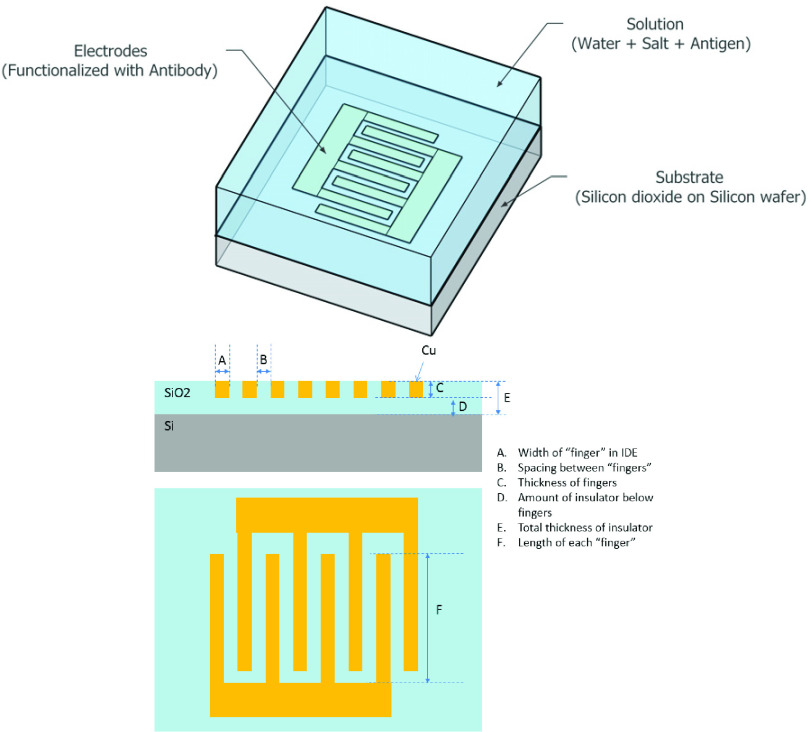
Schematic of the sensor’s electrode configuration modeled in this study.

**FIGURE 2.**
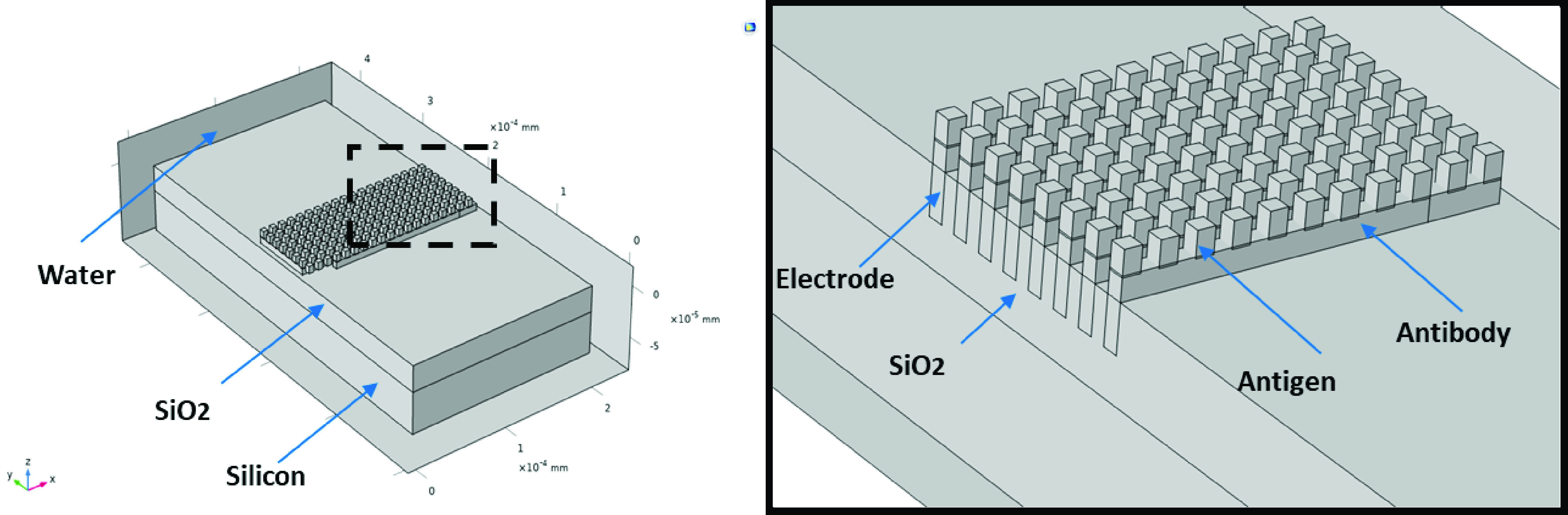
Schematic showing the geometry of the electrode area with attached antibody layer and individual antigens (simplified as block structures).

The sensor was simulated based on a nanoscale damascene process in which the pattern is etched into a dielectric (SiO_2_), filled with the metal electrode layer (in this case, Cu), and planarized using chemical mechanical planarization (CMP). The general format of the simulated sensor in this study is shown in Figure 1. The electrodes have a width of A, length of F, and spacing of B. An insulating layer D of SiO_2_ was placed underneath the sensor electrodes while the total thickness of the SiO_2_ insulator is represented by E. Table 1 lists the starting dimensions that were used in the simulations, while Figure 2 illustrates the geometry of the sensor.

**TABLE 1:** Sensor Electrodes Dimensions Used for the Simulated Model

Parameter	Dimension
A	50 nm
B	50 nm
C	130 nm
D	120 nm
E	250 nm
F	1400 nm

**FIGURE 3.**
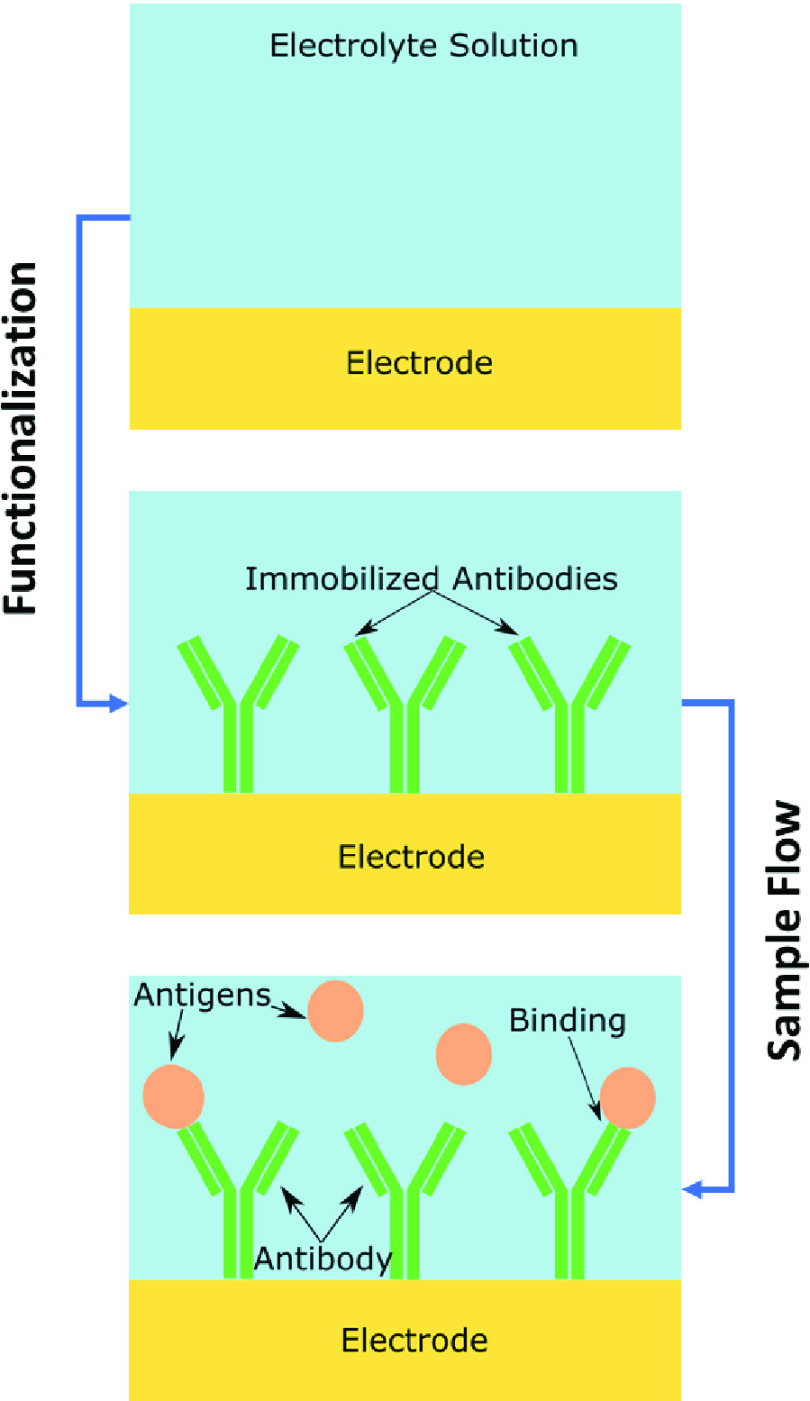
Example showing functionalization of the electrode surface and subsequent immobilization of antibodies, followed by antigen binding.

The sensor’s electrodes were simulated as the capacitive element in an RC circuit in COMSOL and the relative geometry of the electrodes was modulated and assessed for electrical impedance changes within the context of antibody-antigen based biosensing. In this case, protein-based antigens were placed on the surface of the electrodes (linked via a thin attachment layer), followed by addition of an antibody (IgG) layer (Figure 3). This simulates a common biosensing approach, in which bloodborne antibodies react with the sensor to indicate an immune response to a pathogen. Using this approach, small changes in the number of antibodies bound to antigens on the electrodes surface should impart a measurable change in impedance.

For simulation in this work, antigens and antibodies dimensions and parameters are introduced in Table 2 [23]–[25]. The protein is simulated as a thin layer that coats the bare electrodes, while the IgG is represented as an array of blocks that will bind with the protein coat. The number of blocks in the array can be modified to simulate the total number/concentration of antigens and antibodies binding to the electrodes.

**TABLE 2:** Antigen and Antibody Dimensions and Parameters

IgG	$50 \times 50 \times 70 \text{ nm}$
Protein	$50 \times 70 \text{ nm}$
Electric Conductivity (Sigma)	0.000000825 S/m
Relative Permittivity (Epsilon)	10

A Nyquist plot (imaginary component of the impedance $\vert \text{Z}\vert $ vs. real component of $\vert \text{Z}\vert$) shows any change in the impedance when protein or IgG is added to the electrodes’ surface. The assumption is that increasing the amount of dielectric on the electrode creates additional resistance (and possibly capacitance) and that should cause an increase in the diameter of the semi-circle (absolute value of impedance level) for the measurement.

## III. COMSOL Simulation Results and Discussion

COMSOL was used to simulate the sensor to verify the first two parts of this paper; first, the impedance/virsus detection correlation and second the how changes to design would affect the impedance measured at different frequencies. Nyquist plots were used to visualize changes in impedance under these different conditions. It was expected that the impedance would increase with more protein added.

### A. Impedance/Protein Detection Correlation Proof of Concept

**FIGURE 4.**
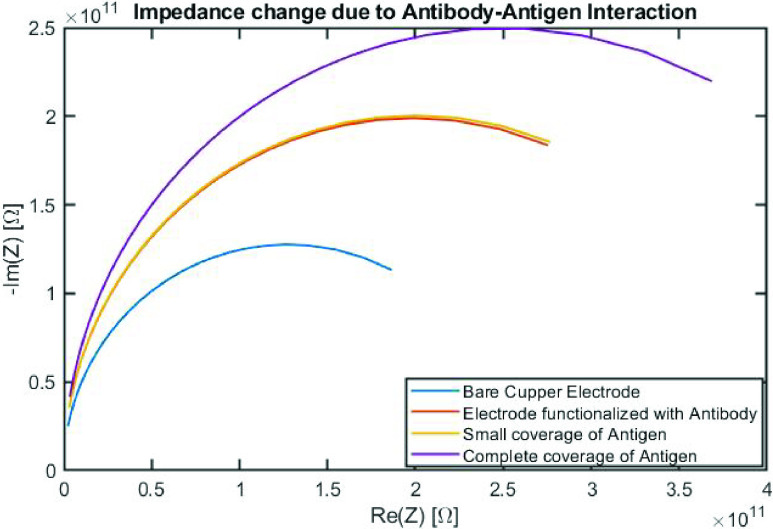
COMSOL simulation results showing the effect of adding antibody and antigen (protein) layers to the sensor on expected impedance $\vert \text{Z}\vert $.

COMSOL was first used to simulate three different scenarios for nanoscale sensor. The first scenario simulated bare electrodes with no protein-based antigen or IgG above the copper electrodes. The second scenario simulated addition of the antigen coating layer above the copper electrodes but with no IgG binding. The third scenario explored IgG binding to the antigen coating layer. Figure 4 shows the simulation-based impedance response for each of these scenarios. As shown, adding more protein materials above the sensor electrodes causes an increase in the impedance as expected. Moreover, an addition of a small amount of antibody (IgG) above the antigen layer (simulating an antibody-antigen binding response) results in a small, but insignificant change to the impedance. However, almost complete coverage of antigen binding to antibody results in a significant increase to the impedance.

### B. The Sensor Dimensions Study

**FIGURE 5.**
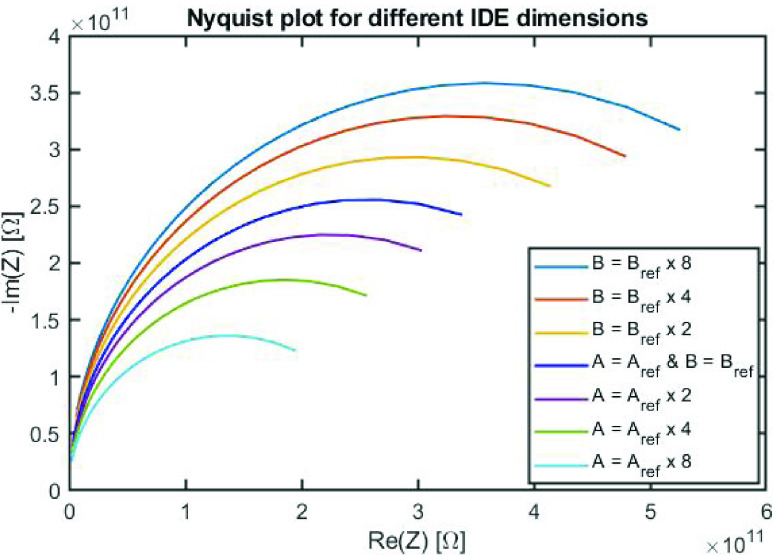
Changes in impedance as a function of modulating electrodes dimensions.

The dimensions of the electrodes were also studied to determine if these dimensions would have an effect on impedance. The electrode width (A) and the spacing between electrodes (B) were both varied. Nyquist plots were then generated from simulations of these configurations, using a constant amount of antibody and antigen. As illustrated in Figure 5, the sensor’s electrodes width (A) was increased to $2\times $, $4\times $, and $8\times $ the initial reference width. The resulting impedance values decreased with increasing electrode width. When the spacing between the electrodes was modified ($2\times $, $4\times $ and $8\times $ the initial reference), the resulting overall impedance of the sensor increased.

## IV. Prototype Development

**FIGURE 6.**
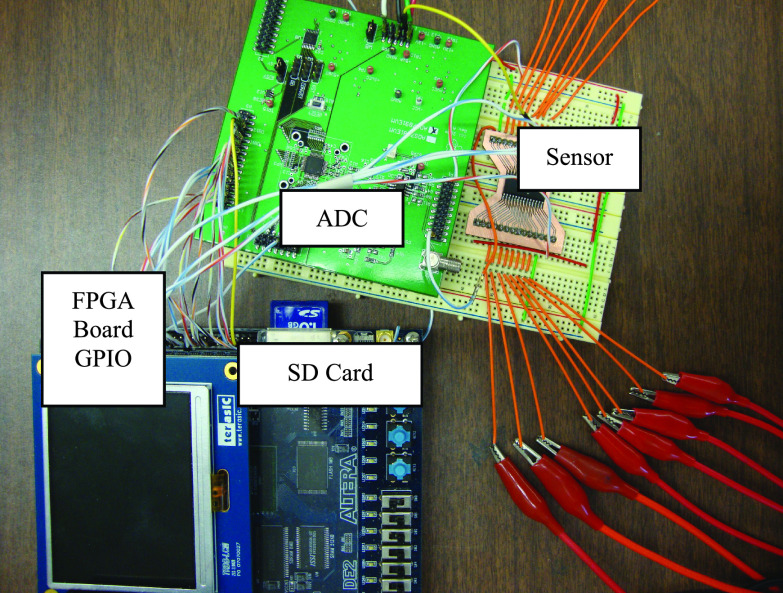
FPGA based DSP to acquire and process signals from sensor.

The third part of this paper presents the home healthcare device/system prototype and its evaluation. Field Programmable Gate Array (FPGA) is used as the main platform to process and analyze the acquired signals from the sensor. As shown in Figure 6, the sensor is fabricated on a small printed circuit board (PCB) which is connected to Texas Instruments (TI) [26] data acquisition card with analog to digital converter (ADC) to measure both the voltage and the current via a burden resistance [26]. The ADC output is connected to the GPIO pins of the FPGA as shown in Figure 6. In the proposed prototype, an Altera Cyclone V FPGA is used. The signal processing is performed in Verilog hardware description language and the calculated impedance based on the sample rate is stored on the attached SD card for future analysis and inspection. So, all readings can be accessed later by the patient’s physician when needed.

The prototype’s evaluation will be done via an experiment to compare the readings of the impedance in two situations; first, with no protein on the sensor (i.e with bare copper); second, with the existence of the protein which represents the virus detection or at least change in the human cell. Moreover, when a change is reported by the FPGA monitoring system, a signal must be triggered as an indication to the change in the impedance. Once the healthcare monitoring system reports the change in the impedance, the patient must seek help, otherwise, no need to regular visits to the healthcare facilities.

The protein used in this research is Dulbecco’s Modified Eagle’s medium (DMEM) which is considered one of the more common and less complex in comparison to enriched media which are utilized for more specialized cell types and are the basis for more unique serum-free media formulations. It contains a higher concentration of amino acids and vitamins, as well as additional supplementary components [27].

## V. Prototype Design and Evaluation

**FIGURE 7.**
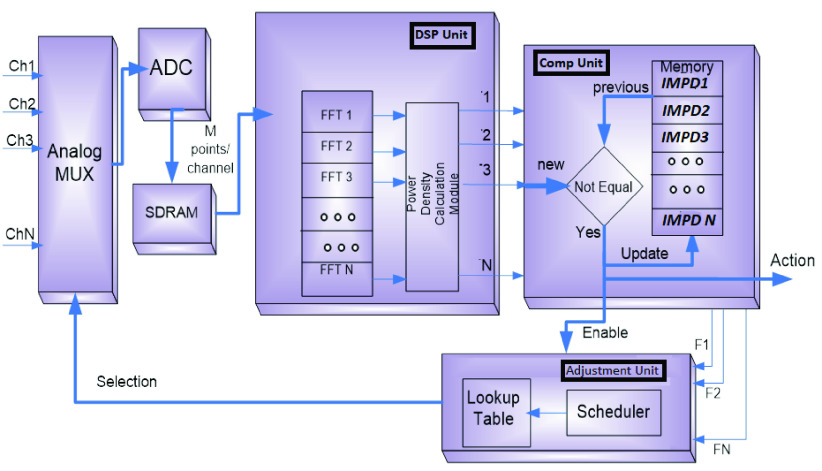
Mux, DSP, Comparator, Adjusting Units.

The proposed design, shown in Figure 7, allows connecting more than one sensor, each sensor will be connected to one channel of a multiplexer which is connected to a single ADC to save and reduce the circuit size and cost. It is proposed to have a multiplexed single ADC rather than having a dedicated ADC for each sensor especially for this type of measurement; since high sampling rate is not a must. Once the ADC captures the signal from the sensor, it is given to SRAM on the FPGA which will act as an array to feed the signal samples to the Digital Signal Processing (DSP) unit inside the FPGA. After the DSP detects the impedance, it reports the values to the comparator unit which stores all values of impedances over time in a memory to be written into the SD card. Meanwhile, a very straightforward comparison operation between the current impedance reading and the previous one is performed via the comparator unit. If no change in the impedance, so there is no need to take any actions, otherwise, if there is a significant change in the impedance, a signal (*Action* signal shown in Figure 7) will be triggered. This signal can be just as simple as turn on an LED or it can be something like calling the patient’s physician. In the current prototype, it is just coded to just turn on one of the FPGA’s LEDs. Last but not least, the adjustment unit is developed to adjust the multiplexer’s scheduler to add more samples from a certain channel over other channels when needed for more accurate readings.

**FIGURE 8.**
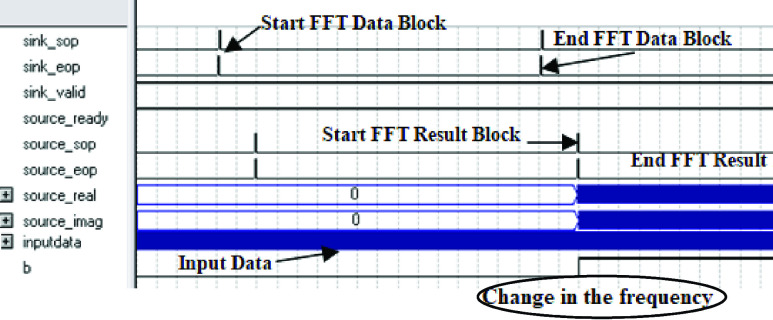
FPGA FFT Unit results show the change in the impedance flag.

It is reported and confirmed that the values of the impedance are increased when the sensor is covered in protein/cell media which matches the same findings from the COMSOL simulation. Moreover, another test is performed to verify the ability of the developed system to detect the change in the impedance and trigger a signal as a result of this change. Fast Fourier Transform (FFT) unit is designed and developed in the FPGA to detect the change in the frequency which indicates the change in the reactance based on Xc = 1/wC. In Figure 8, sensor readings are sampled and block of samples is taken each predetermined period of time as shown in the figure as “*Start FFT Block* and *End FFT Block*”. It takes two cycles to start getting the first FFT result. After processing the whole block, if there is a change in the frequency which represents a change in the reactance/impedance, a flag will be triggered as shown in the figure below “*Change in the frequency*”.

## VI. Conclusion

Early diagnosis of viral infections is critical in patients’ lives. A continues health monitoring may be required to detect viruses in an early stage. In this research, a novel sensor and healthcare monitoring system is modeled, simulated, developed, and tested to detect viruses by detecting the change in the impedance. This paper has three main components, first, COMSOL is used to develop a model to prove the main concept of the paper which is there is a strong correlation between the impedance and virus detection or cell behavior change. The model predicts increasing impedance during functionalization of electrodes with antibodies and after antigen binding steps. The second component of this paper is to understand how nanoscale electrode dimensions such as size and spacing would affect biosensing assay (antibody-based affinity binding of a protein antigen). In the last component of this paper, FPGA-based signal processing system is developed to acquire the current and voltage readings of the sensors over time. This healthcare monitoring system is used to continuously monitoring a patient’s condition and reports any changes in the impedance readings which represents virus detection or at least change in the cell’s behavior.

The proposed sensor model is simulated, tested and verified via COMSOL and the FPGA prototype is tested and it verified the COMSOL model. This work reports that the proposed sensor can be used to detect viruses via detecting a change in the impedance.
